# Invasive and Noninvasive Multimodal Bedside Monitoring in Subarachnoid Hemorrhage: A Review of Techniques and Available Data

**DOI:** 10.1155/2013/987934

**Published:** 2013-03-31

**Authors:** Baback Arshi, William J. Mack, Benjamin Emanuel

**Affiliations:** ^1^Department of Neurology, University of Southern California, Health Sciences Campus, CHP 209 D, Mail Code 9207, Los Angeles, CA 90089-9207, USA; ^2^Department of Neurosurgery, University of Southern California, Health Sciences Campus, CHP 209 D, Mail Code 9207, Los Angeles, CA 90089-9207, USA

## Abstract

Delayed-cerebral ischemia is a major cause of morbidity and mortality in the setting of aneurysmal subarachnoid hemorrhage. Despite extensive research efforts and a breadth of collective clinical experience, accurate diagnosis of vasospasm remains difficult, and effective treatment options are limited. Classically, diagnosis has focused on imaging assessment of the cerebral vasculature. Recently, invasive and noninvasive bedside techniques designed to characterize relevant hemodynamic and metabolic alterations have gained substantial attention. Such modalities include microdialysis, brain tissue oxygenation, jugular bulb oximetry, thermal diffusion cerebral blood flow, and near-infrared spectroscopy. This paper reviews these modalities and examines data pertinent to the diagnosis and management of cerebral vasospasm.

## 1. Introduction

Delayed-cerebral ischemia (DCI) is a major cause of morbidity and mortality in the setting of aneurysmal subarachnoid hemorrhage (aSAH) [1]. Early diagnosis and effective treatment of cerebral vasospasm remain considerable challenges. Neurologists, neurosurgeons, and intensivists utilize multiple advanced diagnostic imaging techniques and examine a broad spectrum of physiologic parameters in attempts to identify reversible cerebral ischemia following aSAH. These modalities provide important data that guide treatment decisions and influence management protocols. Nonetheless, vasospasm related morbidity and mortality remain exceedingly high. Over the past twenty years, efforts have centered on identifying metabolic substrates relevant to the pathogenesis of cerebral vasospasm. As a result, intracerebral monitoring has given rise to a new, multimodal discipline providing specialists, a wide a variety of novel biomarkers potentially relevant to the diagnosis and management of delayed cerebral ischemia. This paper reviews both invasive and noninvasive multimodal bedside monitoring strategies and examines data pertinent to the diagnosis and management of cerebral vasospasm.

## 2. Methods

An extensive literature search through PubMed medical database through July 2012 was conducted using combinations of the keywords “aneurysmal subarachnoid hemorrhage,” “vasospasm,” “microdialysis,” “brain tissue oxygenation (Licox, Integra Neurosciences),” “jugular bulb oximetry,” “thermal diffusion cerebral blood flow (Hemedex, Hemedex Inc.),” and “near-infrared spectroscopy.” All titles and abstracts identified were reviewed. Additional articles were identified from the reference lists of the selected manuscripts. Articles in all languages were eligible. Inclusion was restricted to reports containing original data from studies examining microdialysis, brain tissue oxygenation, jugular bulb oximetry, thermal diffusion cerebral blood flow, or near infrared spectroscopy in the setting of aSAH. Trials incorporating imaging to validate the detection of delayed cerebral ischemia with invasive intracerebral monitoring as well as trials that did not have imaging correlates were included. Articles that lacked original data including review articles, meta-analysis, and case reports were excluded. Trials evaluating multimodal modalities in patients with only traumatic brain injury (TBI) were also excluded.

## 3. Results

### 3.1. Microdialysis

Invasive microdialysis monitoring enables assessment of regional cerebral metabolic physiology and provides biomarkers for clinical correlation [2]. Extracellular pyruvate (166 ± 47 *μ*mol/L), lactate (2.9 ± 0.9 mmol/L), glucose (1.7 ± 0.9 mmol/L), glutamate (1.6 ± 1.6 *μ*mol/L), glycerol (82 ± 44 *μ*mol/L), and lactate/pyruvate ratio (23 ± 4) have been studied, and reference ranges established [3].

Studies have suggested that extracellular alterations in glutamate, lactate, lactate/pyruvate ratio, and glycerol are associated with delayed cerebral ischemia following aSAH [4–7]. Recent attempts have been made to correlate metabolic changes with findings on TCD, computed tomography (CT), and positron emission tomography-computed tomography (PET CT). A 2001 study of sixty patients by Unterberg et al. demonstrated that microdialysis values had a higher specificity (0.89, 95% CI 0.78–1) for delayed cerebral ischemia in symptomatic vasospasm patients than did TCD (0.63, 95% CI 0.46–0.8) or digital subtraction angiography (0.53, 95% CI 0.35–0.7) [8].

A study of forty aSAH patients conducted by Kett-White et al. correlated microdialysis levels with clinical presentation grade. The authors demonstrated that unfavorable World Federation of Neurosurgical Societies (WFNS) scores were associated with higher extracellular glutamate, lactate, and lactate/pyruvate ratio.

Patients presenting with a WFNS grade 1, 2, or 3 demonstrated mean levels of extracellular glutamate (3.5 *μ*mol/L, 95% CI 2.1–4.9), lactate (2.3 mmol/L, 95% CI 1.7–2.9), and lactate/pyruvate ratio (20.8, 95% CI 16.6–25) that were significantly lower than those of WFNS grade 4 or 5 patients ( glutamate 5.5 *μ*mol/L; 95% CI 1.6–9.4; lactate 3.7 mmol/L; 95% CI 2.9–4.5; and lactate/pyruvate ratio 32.0; 95% CI 25.7–38.3) [9]. These findings have been corroborated by several independent studies [4–7]. 

Studies have also correlated extracellular cerebral microdialysate values with findings on diagnostic imaging. Prior studies have shown that PET can reliably demonstrate reduction in cerebral blood flow secondary to vasospasm [10]. Sarrafzadeh et al. demonstrated that a reduction in regional cerebral blood flow (rCBF), as determined by ^15^O-H_2_O PET, is best reflected by elevations in extracellular glutamate and glycerol levels [6]. The authors utilized the following microdialysis parameters for detection of cerebral ischemia: glutamate > 5 *μ*mol/L, lactate > 4 mmol/L, lactate/pyruvate ratio > 25, glycerol > 70 mmol/L, and reduced rCBF < 20 mL*·*100 g^−1^ *·*min^−1^ [6].

### 3.2. Brain Tissue Oxygenation

Brain tissue oxygenation (PbtO_2_) catheters quantify brain tissue oxygen pressure in focal cortical regions as a surrogate for alterations in oxygen delivery or cerebral demand [11]. Studies have demonstrated a predisposition for cerebral ischemia when PbtO_2_ levels were <10 mm Hg [12, 13]. A large trial conducted by Jaeger et al. evaluated PbtO_2_ monitoring in the setting of cerebral vasospasm. The authors demonstrated that PbtO_2_ could accurately predict impaired vascular autoregulation. In a study of sixty-seven patients, there was significant differences in PbtO_2_ levels between patient cohorts with (20.8 mm Hg ± 5.0) and without (23.9 mm Hg ± 5.8) (*P* < 0.06) evidence of cerebral infarction [14]. Of note, the PbtO_2_ values differ from those reported by Meixensberger et al. and Väth et al., perhaps owing to sampling at earlier posthemorrhage time points including days 1–4 [12, 13]. Nevertheless, trends indicate that lower PbtO_2_ is suggestive of cerebral ischemia. In the patients without evidence of infarction on CT scan, maximum MCA velocities were 160 ± 46 cm/s, while values in those with evidence of cerebral infarction were 172 ± 46 cm/s (*P* = 0.32) [14].

In a twenty-three-patient study, Cerejo et al. successfully correlated PbtO_2_ measurements with evidence of vasospasm on TCD (MCA > 120–180 cm/s, ICA > 130 cm/s, ACA > 120 cm/s, PCA > 90 cm/s, Basilar > 100 cm/s, and Vertebral > 90 cm/s) [15]. Postoperative vasospasm patients had significantly lower PbtO_2_ values of 5.4 mm Hg versus 11 mm Hg (*P* = 0.002) [15]. These results corroborate findings by Meixensberger et al. suggesting that PbtO_2_  <  10 mm Hg is suggestive of regional cerebral ischemia [12]. The trend demonstrated that cerebral hypoxia was associated with poor outcomes. 

A study by Meixensberger et al. sought to utilize intracranial pressure (ICP), cerebral perfusion pressure (CPP), and PbtO_2_ as complementary tools in the guidance of vasospasm therapy [16]. However, the investigation did not establish early PbtO_2_ levels as an accurate predictor of outcome [16]. PbtO_2 _ measurements allowed for detection of cerebral infarction, but not prediction of cerebral ischemia. The lack of predictive value rendered PbtO_2 _ less useful than ICP in guiding clinical therapies [16]. Due to sensitivity for symptomatic ischemia, hypertensive, hypervolemic, and hemodilution (HHH) therapy was guided by ICP values, rather than PbtO_2_. Ramakrishna et al. showed PbtO_2_ to be higher in aSAH survivors, 33.94 mm Hg ± 2.74, when compared to nonsurvivors, 28.14 mm Hg ± 2.59 (*P* = 0.05) [17]. The threshold for cerebral hypoxia in this study was PbtO_2 _ < 15 mm Hg in this study. Despite a relatively high threshold, compromised PbtO_2 _correlated with 1-month mortality following SAH [17].

While data can be extrapolated, and information can be gained from the results of large TBI trials, further studies are required to validate the routine use of PbtO_2_ in the setting of aSAH.

### 3.3. Jugular Bulb Oximetry

Jugular bulb oximetry has been used to monitor jugular venous desaturation. It is hypothesized that low jugular venous oxygen levels and anaerobic metabolism are associated with poor neurologic outcome [18]. Heran et al. examined the utility of increased cerebral oxygen extraction (AVDO_2_) and resultant jugular venous desaturation, as evidenced by jugular bulb oximetry, in predicting cerebral vasospasm [19]. Of the 14 patients studied, those who developed vasospasm demonstrated changes in jugular venous oxygen saturation. In a matched analysis, the vasospasm cohort had a baseline AVDO_2_ of 27.6%, while the nonvasospasm patients had an AVDO_2_ of 40.0% [19]. 

### 3.4. Thermal Diffusion Cerebral Blood Flow

Thermal-diffusion flowmetry utilizes cerebral blood flow measurements to predict vasospasm. Probes are placed in “at-risk” white matter regions through intracranial bolts and are used to measure regional cerebral blood flow [20]. Although most data is generated from TBI patients, a study by Vajkoczy et al. demonstrated utility in the setting of aSAH. In a 14-patient study, Hemedex monitors appeared effective in predicting cerebral vasospasm as defined by angiography, Xe-enhanced CT scans, and clinical parameters [21]. In vasospasm patients, thermal-diffusion flowmetry (TD-rCBF) measurements decreased from 21 ± 4 to 9 ± 1 mL/100 g/min, while the control group maintained cerebral blood flow values of 25 ± 4 mL/100 g/min. The authors identified a TD-rCBF value of 15 mL/100 g/min as an appropriate threshold for the diagnosis of symptomatic vasospasm [21].

### 3.5. Near-Infrared Spectroscopy

Near-infrared spectroscopy (NIRS) allows for monitoring of cerebral blood flow by local hemoglobin oxygen saturation (rSO_2_) measurement [22–24]. Probes are noninvasively secured to the scalp with adhesive pads, allowing for prolonged monitoring. The majority of studies to date have employed infrared light at uniform wavelengths of 730 nm, 810 nm, and 850 nm [22–24]. Mutoh et al. continuously monitored patients and compared rSO_2 _to TCD, MR angiography, and stable technetium-99 m hexamethylpropyleneamine oxime single photon emission computed tomography (SPECT). Decreased rSO_2_ was associated with cerebral vasospasm according to MCA TCD flow velocities > 120 cm/s, Lindegaard ratio > 3, and/or imaging criteria [22]. Specifically, affected patients demonstrated a 24% ± 4% ipsilateral decrease in rSO_2_ compared to measurements from the contralateral, unaffected hemisphere [22]. 

A further study comparing NIRS performance to daily TCD measurements demonstrated early prediction of vasospasm [25]. In fact NIRS showed an abrupt decrease in rSO_2_ between days 5–9 after aSAH, which correlated with vasospasm on cerebral angiography [25]. Monitoring capability has improved via reductions in hemoglobin concentration variability and adjustments controlling for effects attributable to cerebrospinal fluid and skull thickness [26–28]. 

## 4. Conclusion

Efforts focused on the application of novel neurointensive monitoring techniques in the setting of cerebral vasospasm have yielded encouraging results. Multiple international sites have contributed to an expanding cache of research that has generated significant interest within the academic community. While these modalities have not yet gained universal acceptance or widespread implementation, they hold real promise for future application. Large-scale prospective trials are needed for validation. Comparative analyses among the proposed modalities would help determine relative effectiveness in a range of clinical settings. Considerable research efforts have focused on Microdialysis, PbtO_2_, and near-infrared spectroscopy, yet data is less abundant for Jugular bulb oximetry and thermal diffusion cerebral blood flow. While invasive neuromonitoring may possess diagnostic utility in the setting of delayed cerebral vasospasm, additional research is needed to determine the efficacy of the different monitoring modalities and refine our understanding of their clinical applications.
